# Early versus late surgical stabilization of severe rib fractures in patients with respiratory failure: A retrospective study

**DOI:** 10.1371/journal.pone.0216170

**Published:** 2019-04-25

**Authors:** Ying-Hao Su, Shun-Mao Yang, Chun-Hsiung Huang, Huan-Jang Ko

**Affiliations:** 1 Department of Orthopedics, National Taiwan University Hospital Hsin-Chu Branch, Hsin-chu City, Taiwan; 2 Division of Thoracic Surgery, Department of Surgery, National Taiwan University Hospital Hsin-Chu Branch, Hsin-chu City, Taiwan; 3 Division of Trauma Surgery, Department of Surgery, National Taiwan University Hospital Hsin-Chu Branch, Hsin-chu City, Taiwan; Baylor College of Medicine, UNITED STATES

## Abstract

**Introduction:**

The timing of surgical stabilization of rib fractures remains controversial. We hypothesized that early surgical stabilization (within 3 days of injury) can improve clinical outcome in patients with severe rib fractures and respiratory failure. The aim of this study was to analyze the impact of early surgical stabilization of rib fractures on the perioperative results, clinical outcomes, and medical costs of patients with severe rib fractures and respiratory failure.

**Methods:**

This was a retrospective comparative study based on a prospectively collected database at a single institute. Patients with severe rib fractures and respiratory failure who underwent surgical stabilization were classified into early (within 3 days of injury) and late (more than 3 days after injury) groups. Outcome measures included operation time, duration of mechanical ventilation, intensive care unit stay, hospital stay, complication rate, mortality rate, and medical cost.

**Results:**

A total of 33 patients were enrolled (16 and 17 in the early and late groups, respectively). The demographics, trauma mechanism, associated injuries, and severity of trauma were comparable in both groups. The early group had significantly shorter duration of mechanical ventilation (median 36 vs. 90 hours, *p* = 0.03), intensive care unit stay (median 123 vs. 230 hours, *p* = 0.004), and hospital stay (median 12 vs. 18 days, *p* = 0.005); and lower National Health Insurance costs (median 6,617 vs. 10,017 US dollars, *p* = 0.031). The early group tended to have lower rates of morbidity and mortality, but the difference was not statistically significant.

**Conclusion:**

Early surgical stabilization of rib fractures in selected patients may significantly shorten their duration of mechanical ventilation, and intensive care unit and hospital stays, while incurring less medical costs.

## Introduction

Blunt chest trauma often causes rib fractures, which may be accompanied by hemothorax, pneumothorax, and intrapleural and intrathoracic lesions. The patients suffer from severe chest wall pain, deformity, and subsequent pneumonia and/or respiratory failure (RF).[1] To this date, three randomized controlled trials (RCTs) have shown that surgical stabilization of rib fractures (SSRFs) for flail chest is superior to conservative treatment.[2–4] Reviews and meta-analyses showed that SSRF can reduce the duration of mechanical ventilation (DMV), intensive care unit (ICU) length of stay (LOS), and hospital LOS.[5–7] It also decreases the rate of pneumonia and tracheostomy. The surgical indications for SSRF have been outlined by several studies.[8–10] Patients with flail chest, multiple rib fractures, RF, intractable pain after conservative treatment, loss of lung function, and chest wall deformity are candidates of SSRF.

Recently, the timing of SSRF has shifted to the early stage.[11] However, there remains a paucity of data on the optimal timing of SSRF. In the 3 aforementioned RCTs, SSRF was performed within 2–5 days of intubation in patients with recognized RF.[2–4] The procedure provided significant benefit over conservative treatment. Althausen et al. described a positive correlation between hospital LOS, ICU LOS, DMV and time to operation in patients undergoing SSRF.[12] The SSRF consensus statement suggests that SSRF should be performed within 72 hours of injury.[10] Pieracci et al’s multicenter trial demonstrated that patients undergoing SSRF within 24 hours of admission had more favorable outcomes than those that underwent SSRF within 4–10 days of injury.[11]

We hypothesized that early SSRF (within 3 days of injury) in patients with severe rib fractures and RF can shorten the hospital course and improve the clinical outcomes with less medical costs. The aim of this study was to compare the demographics; perioperative findings; clinical outcomes, and the factors associated with them, of patients undergoing early versus late SSRF.

## Methods

### Study population

The study population consisted of patients 18-years-old or older who met our institution’s criteria for undergoing SSRF. All patients were admitted through the emergency department and underwent complete evaluation including 3-dimensional computed tomography of the chest. Our indications for SSRF were (1) flail chest, (2) 4 or more rib fractures with bicortical displacement, (3) rib fractures with RF, or (4) intractable pain after conservative treatment (pain Visual Analogue Scale >6).[3,8–10] The upper ribs (1^st^ and 2^nd^) and floating ribs (11^th^, 12^th^) were not fixed. Patients with severe head injury with (Glasgow coma scale <14) were excluded. RF status included recognized and impending RF. Recognized RF was defined as pre-existing RF with intubation and mechanical ventilation (MV) prior to SSRF. Impending RF was defined as a continuously deteriorating respiratory status which requiring non-invasive ventilation with at least one of following parameters: (1) respiratory rate >25 times per minute, (2) pulse oximetry <92%, (3) deteriorated pulmonary toilet, or (4) clinically judged by the physician, which were modified from the indication for non-invasive ventilation.[13] If the patients had sustained associated fractures of the lower limbs or pelvis, an orthopedic surgeon on duty (other than the orthopedic surgeon that would perform the SSRF) was consulted. If the patients had sustained concurrent subarachnoid hemorrhage with a Glasgow coma scale >14, the neurosurgeon on duty was consulted.

### Study design

This was a retrospective comparative study based on a prospectively collected database at our institute. The charts and operative records were reviewed. Patients with flail chest and 4 or more rib fractures who also had RF were enrolled. The patients were divided into early (within 3 days of injury) and late (>3 days) SSRF groups. Those with injuries that required emergent surgery, such as massive hemothorax or injury to the trachea or bronchus, were excluded. The interval (days) between the operation and the date of injury was used to define the early and late groups. Most patients could only remember the date of injury. Furthermore, some of our SSRF patients were transferred from other institutes with impending RF status, and their decompensated RF status was different from their initial status after sustaining the blunt chest trauma. Database evaluation and charts were reviewed to extract perioperative and clinical results. The perioperative results investigated included operation time (from skin incision to wound closure), intraoperative findings, and additional surgeries. The primary endpoints were DMV and ICU LOS. The secondary endpoints were duration of chest tube insertion, hospital LOS, pneumonia rates, transfer to a respiratory ICU, and death.

Evaluation of the medicals cost was based on data obtained from the National Health Insurance (NHI) database, which is provided by the Taiwan Government. All the medical costs associated with SSRF were covered by the NHI except for the implants for rib fractures. Total cost included NHI cost, the costs associated with implants for rib and associated limb fractures, meals, and upgrading to a private ward from the general ward. Medical cost was defined as both NHI and total cost and expressed in United States Dollar (USD) after conversion from Taiwan dollar (TWD; USD 1 = TWD 31).

### Surgical technique and patient management

**Fig 1:**
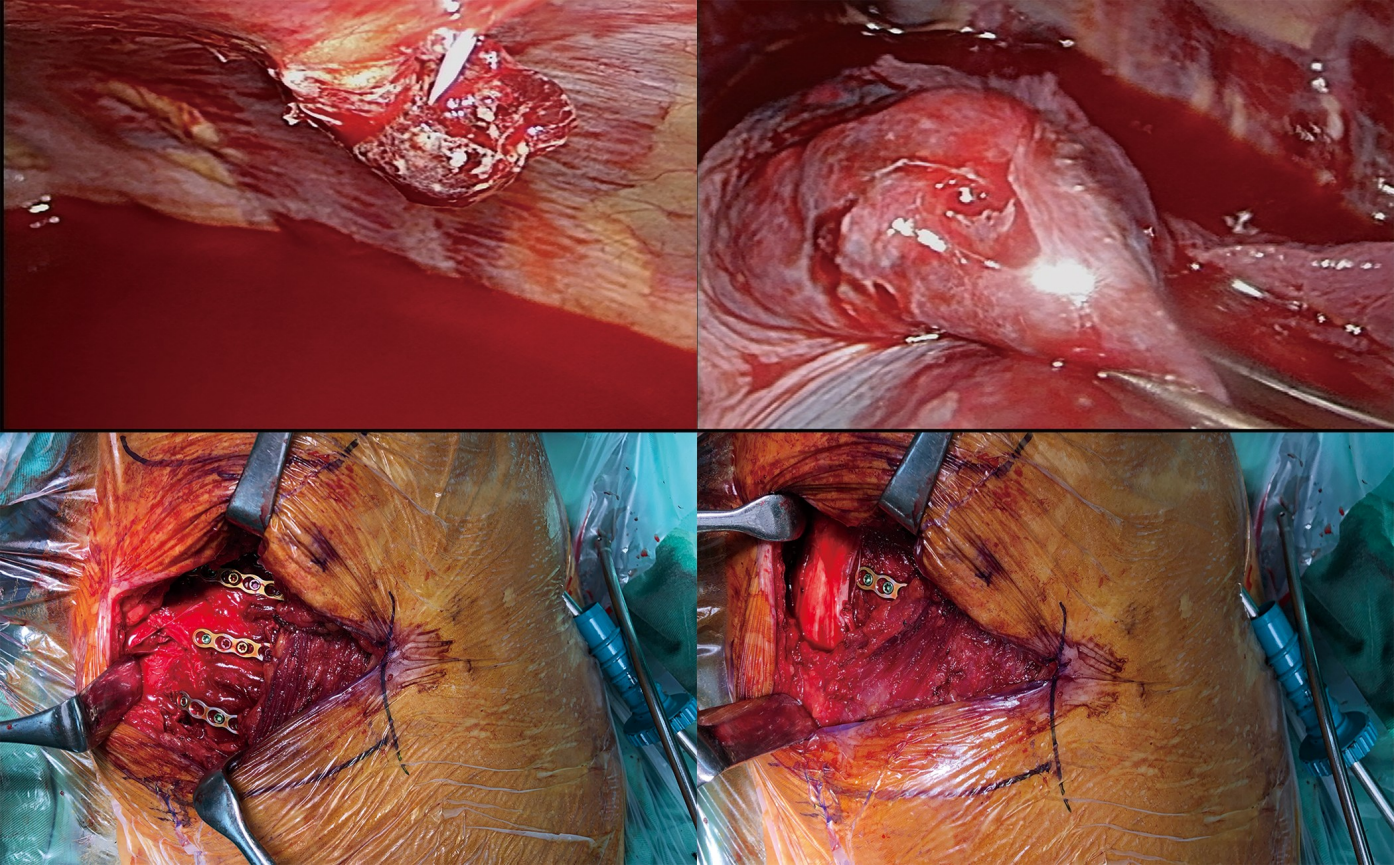
Illustrative case of a patient who underwent surgical stabilization of rib fractures. (A) Video-assisted thoracoscopic surgery (VATS) was utilized to clear the pleural space prior to open reduction internal fixation (ORIF) for rib fractures. The fractured fragment had penetrated through the parietal pleura causing hemothorax. (B) Lung laceration with air leakage was noted and repaired with a linear cutter stapler. (C) The patient underwent combined VATS and ORIF with locking plates through a muscle-sparing approach without thoracotomy. (D) The intercostal and chest-wall muscles were well-preserved after ORIF through this approach.

The surgical team for SSRF was composed of an experienced thoracic surgeon and an orthopedic surgeon.[8] The thoracic surgeon had significant experience with both traditional open thoracotomy approaches and video-assisted thoracoscopic surgery (VATS). The orthopedic surgeon was familiar with ORIF.[8] Our standard surgical method for SSRF is combined VATS and ORIF through a muscle-sparing approach without thoracotomy.[14–17] VATS was utilized to investigate intrapleural and intrathoracic injuries, such as trapped lung[18] or massive hemothorax. In addition, a safe pleural space was created to prevent iatrogenic injury during drilling or manipulation. If the patients had a chest tube, VATS was performed through the thoracostomy wound with a 5 mm, 30-degree thoracoscope. Otherwise, a 1 cm port at the 5^th^ intercostal space along the anterior axillary line was created. During VATS, traumatic adhesions of the lung, and trapped lungs[18] or diaphragm were released from the parietal pleura using the single-port VATS technique. Subsequently, ORIF with universal non-precontoured 2.4- or 3.5-mm metal locking plates (Synthes, Paoli, PA; Zimmer, Warsaw, IN) (Fig 1) was performed.[12,17] After ORIF, VATS was utilized to check for screw penetration through the parietal pleura. If a lung laceration with air leakage was found, a 2^nd^ VATS port was created to perform repair and resection with a linear cutter stapler.[1,19] A chest tube was then placed under direct VATS visualization. If the patient had sustained an unstable sternal fracture, such as a distraction or segmental fracture, simultaneous ORIF of the sternal fracture was performed with a pre-contoured locking plate (SternaLock Blu System; Biomet Microfixation Inc, Jacksonville, FL).[20] After the SSRF, the patients with RF were sent to the ICU with their endotracheal tubes in place. The patients were managed by either the thoracic or trauma /acute care surgeons. The respiratory therapists also participated in management and planned the patients’ weaning protocols.

### Statistical analysis

Descriptive statistics were expressed as counts (percentage) for categorical variables and medians (range) for continuous and ordinal variables. The Shapiro-Wilk test was used to test for normality. The categorical variables in the early and late SSRF groups were compared using the chi-square test or Fisher’s exact test. The continuous and ordinal variables were compared with the Mann-Whitney U test. A *p* value <0.05 was considered statistically significant. The Kruskal-Wallis test was used to analyze the continuous outcome variables. Logistic regression and odds ratios were used to analyze categorical and dichotomous variables. Pearson’s correlation coefficient was used to measure the association between time from injury to operation and continuous variable outcomes. MedCalc software (MedCalc Software, Mariakerke, Belgium) was used for data analysis.

### Ethics approval

The study was approved by the Institutional Review Board of the National Taiwan University Hospital Hsin-Chu Branch (approval #107-004-E). Informed consent was waived due to the retrospective nature of this study. There is no personally identifiable information in this manuscript.

## Results

**Fig 2:**
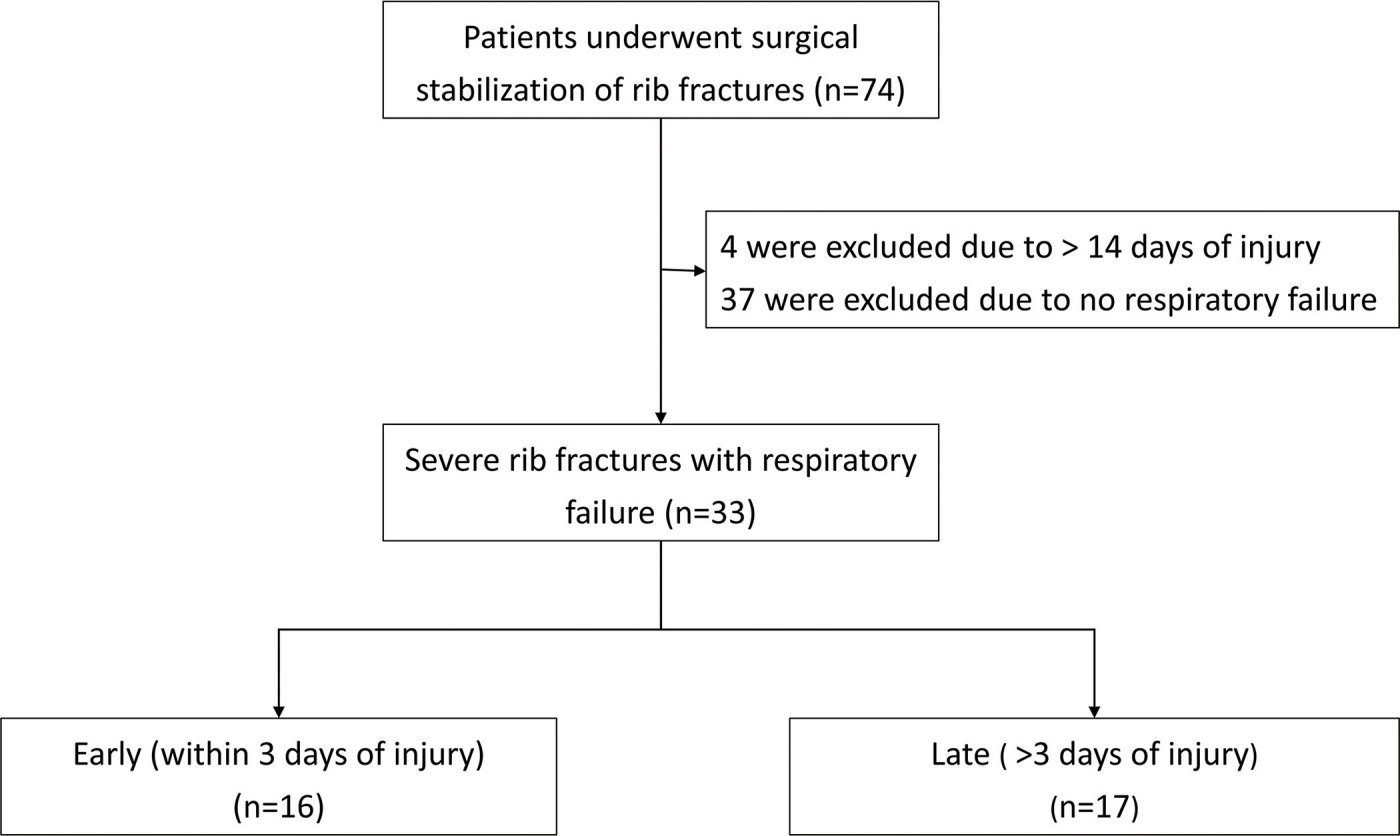
Flow diagram of study selection based on the eligibility criteria.

Between June 2016 and Feb 2018, 74 consecutive patients underwent SSRF at our institute. There were 41 patients who did not meet the criteria of this study: 4 who received SSRF after more than 14 days of injury due to intractable pain and 37 with severe rib fractures but no RF. A total of 33 patients were enrolled and divided into the early (n = 16) and late (n = 17) SSRF groups (Fig 2). The demographics, current smoking status, chronic obstructive pulmonary disease, mechanism of trauma, number of rib fractures, overall number of fractures, associated injuries, RibScore[21], Rib fracture scoring[22], blunt pulmonary contusion 18 score[23], chest abbreviated injury scale, and injury severity score were comparable in both groups (Table 1).

**Table 1:** Group demographics.

	Early (n = 16)	Late (n = 17)	*p*
Age, year, median (range)	62 (19–92)	68 (19–92)	*p* = 0.47
BMI, Kg/m2, median (range)	22.7 (16.8–35.9)	24.2 (17.0–31.7)	*p* = 0.45
Sex, male/female, n	11/5	15/2	*p* = 0.24
Current Smoking, n (%)	2 (12.5)	7 (41.2)	*p* = 0.85
COPD, n (%)	0 (0)	1 (6.3)	*p* = 1.0
Mechanism of the trauma			*p* = 0.44
MSC, n (%)	10 (62.5)	9 (52.9)	
MVA, n (%)	1 (6.3)	1 (5.9)	
Pedestrian, n (%)	2 (12.5)	2 (11.8)	
High-energy fall, n (%)	1 (6.3)	3 (17.6)	
Low-energy fall, n (%)	2 (12.5)	2 (11.8)	
Chest trauma			
Number of fractured ribs, median (range)	5 (4–8)	6 (4–16)	*p* = 0.19
Total number of fractures., median (range)	7.5 (4–16)	9 (4–33)	*p* = 0.36
Flail chest, n (%)	10 (62.5)	11 (64.7)	*p* = 0.44
Hemothorax, n (%)	12 (75.0)	16 (94.1)	*p* = 0.56
Pneumothorax, n (%)	5 (31.3)	7 (41.2)	*p* = 0.84
The RibScore, median (range)	2 (0–4)	3 (0–6)	*p* = 0.38
Rib Fracture Score, median (range)	9.5 (4–16)	12 (4–68)	*p* = 0.22
BPC 18, median (range)	3.5 (1–9)	4 (1–12)	*p* = 0.26
Associated injuries			*p* = 0.57
Clavicle fracture, n (%)	3 (18.8)	6(35.3)	
Scapula fracture, n (%)	5 (31.3)	6(35.3)	
Sternum fracture, n (%)	3 (18.8)	1 (5.9)	
Head injury, n (%)	3 (18.8)	1 (5.9)	
Lower extremity, n (%)	4 (25)	2 (11.8)	
Spinal, pelvic, or visceral injury, n (%)	4 (25)	5 (29.4)	
Chest AIS, median (range)	4 (3–4)	4 (3–5)	*p* = 0.17
ISS, median (range)	20 (9–24)	20 (9–43)	*p* = 0.15

BMI: Body mass index; COPD: Chronic obstructive pulmonary disease; MSC: Motor scooter collision; MVA: Motor vehicle accident; BPC: Blunt pulmonary contusion; AIS: Abbreviated injury scale; ISS: Injury severity score

The timing of the surgery was significantly earlier in the early SSRF group (Table 2). There were no significant differences in operation time, and number of ribs operated on between both groups. In addition, there were no significant differences in the presence of intrapleural lesions, including traumatic adhesions, lung lacerations with air leakage, and pericardial effusion, between the groups. Patients in the early group underwent more additional surgeries, but the difference was not statistically significant. After ORIF, 4 patients in the early SSRF group were found to have active air leakage during re-inflation of the lungs, and repair and resection with a linear cutter stapler was performed. Two patients sustained concurrent sternal fractures and hemopericardium. A pericardiopleural window was created to drain the effusion from the pericardial space. There were 3 patients with unstable sternal fractures in the early SSRF group and 1 in the late SSRF group, all of whom underwent ORIF.

**Table 2:** Perioperative findings.

	Early	Late	*p*
Timing, day	2 (0–3)	6 (4–14)	*p* < 0.001
Length of operation, minutes	105 (69–162)	108 (36–234)	*p* = 0.94
Operated ribs	2 (1–5)	3 (1–6)	*p* = 0.34
Intrathoracic finding			*p* = 0.15
Adhesion, n	2	2	
Lung laceration with air leakage, n	4	0	
Hemopericardium, n	2	0	
Additional surgery			*p* = 0.31
Adhesiolysis, n	2	2	
Repair and resection, n	4	0	
Pericardiopleural window, n	2	0	
ORIF for sternal fracture, n	3	1	

ORIF: Open reduction internal fixation

**Fig 3:**
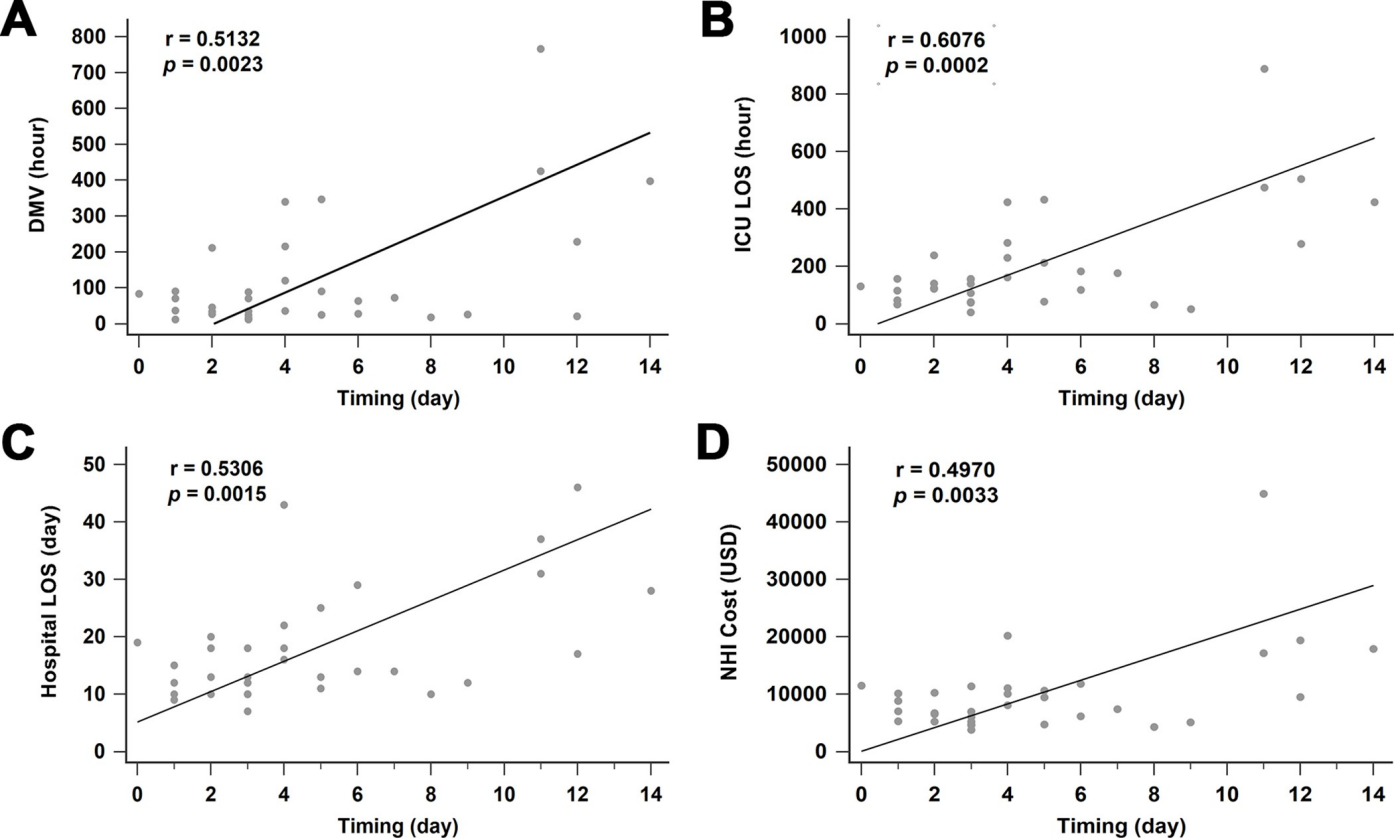
The correlation between surgical timing and related parameters. A: timing and DMV; B: timing and ICU LOS; C: timing and hospital LOS; D: timing and NHI Cost. DMV: duration of mechanical ventilation; ICU: intensive care unit; LOS: length of stay; NHI: National Health Insurance.

The patients in the early SSRF group had significantly shorter DMV, ICU LOS, and hospital LOS (Table 3). There was no difference in the duration of chest tube placement between the groups. No implant-related complications, such as implant failure or surgical site infection, were observed in either group. None of the patients in either group required reintubation after extubation. Pneumonia tended to be less frequent in patients in the early SRRF group, but the difference was not significant. None of these patients elected to undergo tracheostomy. There were no significant differences in the rates of transfer to a respiratory ICU and death between the two groups. The early SSFR group had significantly less NHI medical costs (Table 4). The total cost in the early SSFR group was also less, but not significantly so. Regression analysis showed significant correlations between time from injury to operation and DMV (r = 0.5132, *p* = 0.0023), ICU LOS (r = 0.6076, *p* = 0.0002), hospital LOS (r = 0.5306, *p* = 0.0015), and NHI cost (r = 0.4970, *p* = 0.0033) (Fig 3).

**Table 3:** Clinical results.

	Early	Late	Odds ratio (95% CI)	*p*
DMV, hour	36 (12–212)	90 (18–766)		*p* = 0.03
ICU LOS, hour	123 (40–238)	230 (51–888)		*p* = 0.004
Chest tube, day	7 (4–10)	9 (4–27)		*p* = 0.20
Hospital LOS, day	12 (7–20)	18 (10–46)		*p* = 0.005
Pneumonia, n (%)	2 (12.5)	5 (29.4)	0.34 (0.056 to 2.10)	*p* = 0.25
Transfer to a respiratory ICU, n (%)	0 (0)	1 (5.9)	N/A	*p* = 0.24
Death, n (%)	0 (0)	2 (11.8)	N/A	*p* = 0.10

CI: Confidence interval; DMV: Duration of mechanical ventilation; ICU: Intensive care unit; LOS: Length of stay; ICU: Intensive care unit; N/A: Not applicable.

**Table 4:** Medical cost.

	Early	Late	*p*
NHI Cost*, USD	6,617 (3,805–11,488)	10,070 (4,310–44,913)	*p* = 0.031
Total cost†, USD	11,824 (7,772–22,263)	15,686 (6,112–48,113)	*p* = 0.17

NHI: National Health Insurance; USD: United States Dollar.

* NHI cost does not include the cost of the implants for rib fractures.

† Total cost includes the NHI cost, costs of the rib implants and associated limb fractures, meals costs, and cost of a private ward.

## Discussion

Rib fractures often result from blunt chest trauma and are the main cause of hospital admission. Rib fractures result in pain and disability, and many patients also develop RF, pneumonia, or chest wall deformity. Three previous RCTs showed that SSRF is superior to conservative treatment for patients with flail chest and recognized RF.[2–4] Review articles and meta-analyses also showed that SSRF can reduce ICU and hospital LOS, duration of ventilation, and tracheostomy rate. The indications for SSRF have been well established based on expert consensus.

The timing of surgical intervention is an important factor in most orthopedic trauma, emergency, and acute care operations, and may be an important prognostic factor in these patients. The timing of SSRF is still controversial due to the paucity of data; however, the current trend is toward early SSRF. In the first RCT by Tanaka et al., SSRF was performed within 5 days of MV.[4] In the latest RCT by Marasco et al., SSRF was performed within 2 days of MV.[3] According to the 2017 expert consensus, SSRF should be performed within 72 hours of injury.[10] A multi-center study by Pieracci et al. that compared the outcomes of early, mid, and late SSRF found that SSRF within 24 hours of admission can provide favorable outcomes and significantly shorter operation times. The authors suggested that SSRF should be performed within 24 hours of admission if feasible and applicable.[11] Highly unstable chest wall related-pain and altered respiratory mechanisms would not be corrected until SSRF.

This study based on our limited experience could provide additional information to surgeons that perform SSRF. Patients who were referred to our institute at a late stage due to subsequent and impending RF showed different characteristics from those brought to our emergency department directly. These patients had lung atelectasis, poor pulmonary hygiene, or pneumonia secondary to hypoventilation due to pain. Some of them had poor intake and presented with hypovolemic normotension due to pain. Assessing the interval between the injury and operation could reflect the actual condition of the patients. Both groups had comparable demographics and clinical characteristics. In a comparison of the outcomes of early and late SSRF in patients with RF, the early group had significantly shorter DMV, ICU LOS, and hospital LOS. Early SSRF may stabilize the chest wall, restore chest-wall stability, and reduce chest wall pain. Simultaneous evacuation of hematomas and repair of lung lacerations could reduce the risk of empyema and shorten the duration of chest tube placement. In additions, it could restore the patients’ ventilatory function and enable them to tolerate and receive physical therapy and pulmonary toilet programs earlier. Their intake may return to normal due to improvement of chest wall pain. Earlier weaning off the ventilator can also significantly shorten their hospital course. On the other hand, there was no significant difference in the rates of pneumonia, transfer to a respiratory ICU, or death between the early and late groups. However, this may be due to the small sample sizes in both groups.

In terms of the medical costs, the result of this study showed that early SSRF incurred significantly less NHI expenses due to the significantly shorter duration of MV, ICU LOS, and hospital LOS. Therefore, early SSRF could help reduce governments’ healthcare expenditure. However, the locking titanium plates used for SSRF were not covered by the NHI. Therefore, the total medical cost was higher than the cost to the NHI. In addition, the total cost also included the cost of implants for limb fractures, meal costs, and upgrading to a private ward from the general ward. However, the NHI cost could reflect the actual burden on government expenditure and is more important than the total cost.

In this study, operation time in the early SSRF group was not significantly shorter than in the late SSRF group, in contrast to the results of Pieracci et al.[11] There are several possible explanations; first, our standard approach was combined VATS and ORIF. It took 15–20 minutes to clear the pleural space, evacuate the hemothorax, and irrigate with normal saline. Second, our study population was older and had a lower body mass index than that in Pieracci et al’s study. The adhesion and inflammation in the late SSRF group may be less severe, making it easier to release the adhesion and mobilize the fragments. Third, additional surgeries may be the reason for the increased operation time, more of which were performed in the early SSRF group. When intrapleural and/or lung lacerations with air leakage were present, managing them took 10–20 minutes. In addition to SSRF, 3 patients with unstable sternal fractures underwent ORIF and 2 with traumatic hemopericardium underwent a pericardiopleural window procedure in the early SSRF group. All these factors may explain the lack of a difference in operation time.

The limitations of this study include its retrospective comparative design and single institute setting. Furthermore, the number of cases is relatively small and the interval between the operation and injury date was less precise since patients sometimes found it difficult to recall the date of the accident. The trauma patients studied may have sustained concurrent fractures of the pelvis, spine, or lower extremities; however, the ideal timing for ORIF of displaced fractures of the long bones or pelvis in patients with severe rib fractures and RF remains unclear. Furthermore, the preferred sequence of SSRF and ORIF for unstable fractures of the long bones or pelvis is still controversial.

## Conclusions

The timing of SSRF is an important prognostic factor in the management of patients with severe rib fractures and RF. Patients undergoing early SSRF may have significantly shorter DMV, ICU LOS, hospital LOS, and less NHI costs. When patients with severe rib fractures and RF present to the emergency department, early SSRF should be considered whenever feasible.
